# The Origination of Growth Hormone/Insulin-Like Growth Factor System: A Story From Ancient Basal Chordate Amphioxus

**DOI:** 10.3389/fendo.2022.825722

**Published:** 2022-04-01

**Authors:** Mengyang Li

**Affiliations:** School of Basic Medical Sciences, Qingdao University, Qingdao, China

**Keywords:** amphioxus, GH/IGF system, growth hormone, growth hormone receptor, insulin-like growth factor-I, insulin-like growth factor-I receptor, insulin-like growth factor binding protein

## Abstract

The growth hormone/insulin-like growth factor (GH/IGF) system, also called the pituitary-liver axis, has a somatotrophic role in the body. Although the GH/IGF system has always been regarded as a vertebrate-specific endocrine system, its actual origin remained unknown for a long time. The basal chordate, amphioxus, occupies an evolutionary position between vertebrates and invertebrates. Impressively, most of the members of the GH/IGF system are present in the amphioxus. The GH-like molecule in the amphioxus is mainly expressed in Hatschek’s pit. It functions similarly to vertebrate GH and has a GH receptor-like binding partner. The amphioxus IGF-like peptide shows mitogenic activity and an expression pattern resembling that of vertebrate IGF-I. The receptor of IGF-like peptide and IGF binding protein (IGFBP) have also been demonstrated to exist in the amphioxus. These results reveal the origin of the gene families in the GH/IGF system, providing strong evidence that this system emerged in the amphioxus.

## Introduction

Growth hormone (GH) is a pituitary hormone that has positive effects on the postnatal growth and development of animals. After being secreted into the bloodstream, GH is transported to the target cell membrane by a type of binding protein with high GH affinity (growth hormone binding-protein, GHBP); it then interacts with a specific receptor, the growth hormone receptor (GHR). The interaction between GH and GHR directly triggers the intracellular signal involved in catabolism. Nevertheless, the somatotrophic and anabolic activity of GH predominantly depends on an intermediator defined as insulin-like growth factor-I (IGF-I) (1). GH-targeted hepatocytes can induce the expression of IGF-I in the liver. IGF-I is released into circulation and targets bone and muscle cells. It binds specifically to IGF-I receptor (IGF-IR) located on the cell surface and activates the downstream signal to promote cell proliferation and differentiation. The activity and stability of IGF is regulated by a subgroup of soluble proteins with high IGF- affinity. They are named insulin-like growth factor binding proteins (IGFBPs). These molecules—GH, GHR, IGF, IGFR, and IGFBP—constitute a complicated signaling pathway that is referred to as the GH/IGF system. Owing to the localization of GH and IGF, this system is also denoted as the pituitary-liver axis (2). The GH/IGF system has been found in all taxonomic groups of vertebrates, including agnathans such as lamprey. Since there is limited information on GH/IGF system genes in invertebrates, this system has been regarded as vertebrate-specific for a long time. The cephalochordate amphioxus occupies a nodal position between invertebrates and vertebrates. It appears to be related anatomically and developmentally to ancient vertebrates (3). The amphioxus has the so-called Hatschek’s pit and hepatic caecum, which have been proposed as the homologs of the vertebrate pituitary gland and liver, respectively. Hatschek’s pit was initially identified by Hatschek. It is a dorsal evagination of tall cells from the mouth epithelium and is located to the right of the notochord (4). Similar to the pituitary, Hatschek’s pit originates from the ectoblast. Tjoy and Welsch (5) observed that a type of epithelium cell containing secretory vesicles and secretory grains are distributed in the base of Hatschek’s pit, indicating that it has endocrine activity. A series of immunohistochemical studies further detected positive signals of vertebrate pituitary hormones including luteinizing hormone (LH), follicle stimulating hormone (FSH), and luteinizing hormone-releasing hormone (LHRH) from Hatschek’s pit (6–9). Transcriptions of the pituitary-specific transcription factor Pit-1 and Pax6 have also been detected in the primordium of Hatschek’s pit (10, 11). These results suggest that Hatschek’s pit and the vertebrate pituitary gland are homologous. The amphioxus hepatic caecum is a pouched branch derived from the digestive tube. It has been considered a homolog of the vertebrate liver for a long time (12–14). Studies have shown that the amphioxus hepatic caecum can synthesize liver-specific proteins, including vitellogenin, antithrombin, plasminogen, alanine aminotransferase, glutathione S-transferase, and some acute-phase factors (15–19). In addition, it has been identified that the hepatic caecum regulates glucose metabolism in the amphioxus. Welsh observed that abundant glycogen particles accumulated in the hepatic caecum (20). It was further found that hexokinase and glucose-6-phosphatase (G6Pase), which are essential for glucose metabolism, were specifically expressed in the hepatic caecum (21, 22). The enzymatic activity of glucokinase and G6Pase increased after feeding.

These studies provided sufficient evidence that the amphioxus has organs homologous to the vertebrate pituitary and liver. More importantly, the amphioxus is responsive to the stimulation of vertebrate GH. For instance, the expression of G6Pase in the hepatic caecum was up-regulated by GH (22). This suggested that the specific receptor which can interact with GH and trigger a downstream signal exists in the amphioxus. The members of the GH/IGF system in amphioxus have been gradually identified since the 1990s. In this review, the GH/IGF system and the characterized amphioxus homologues are systemically described. All the evidence suggests that the GH/IGF system emerged in this basal chordate.

## Amphioxus Expresses a Functional GH-Like Molecule

In 1921, Evans and Long first reported that injection of a cow pituitary extract stimulated the growth of rats (23). Subsequent studies showed that hypophysectomy would result in growth arrest (24, 25). In 1944, Li and Evans successfully isolated a peptide hormone from the ox pituitary. It increased the body weight of a hypophysectomised rat (26). Therefore, this hormone was named GH. GH is a multifunctional hormone which regulates growth, development, metabolism, immunity, and internal osmotic pressure (2, 27–29). It is mainly synthesized, stored, and secreted by the pituitary gland. Although GH was initially identified as an endocrine factor, it also acts through an autocrine/paracrine mechanism in some extra-pituitary locations, such as neuronal cells within the central nervous system, epithelial cells, fibroblasts, and some immune cells.

The *GH* genes have been identified in the genomes of all classes of vertebrates. Before the 2010s, it was uncertain whether GH existed in invertebrates. The amphioxus not only has a homolog of the vertebrate pituitary but also responds to stimulation by exogenous vertebrate GH. This implies that GH should be present in the amphioxus. In 2014, Li et al. cloned a *GH-*like (*GHl*) gene from the amphioxus cDNA library (30). This gene contained an ORF encoding a novel type-I helical cytokine of 208 amino acids. Although this cytokine shares low sequence identity with GH, as well as other type-I helical cytokines in vertebrates, phylogenetic analysis showed that *GHl* has a closer genetic relationship with GH. In addition, some conserved amino acid residues essential for binding to GHR are present in the sequence of amphioxus GHl. 3D modeling showed that GHl and human GH share a similar structure, implying that GHl might function like GH. Importantly, amphioxus GHl and GH have similar expression profiles. GHl mRNA and protein have been shown to be highly expressed in Hatschek’s pit, which is homologous to the vertebrate pituitary. Moreover, GHl acted similar to GH *in vivo*. Recombinant GHl protein had a somatotrophic effect in zebrafish and rescued GH-deficient zebrafish embryos. It also promoted sea water adaptation of the amphioxus. Mechanistically, recombinant GHl can interact with GHR and stimulate the expression of IGF-I and osmoregulatory genes, suggesting that GHl exerts its activity through mechanisms similar to those of vertebrate GH (30, 31). These results indicate that a functional GH-like hormone emerged in the amphioxus. 

## The Original Form of GHR and GHBP

GHR is a class-I cytokine receptor that specifically binds to type-I helical cytokines and transduces their signal. It is widely expressed and is most abundant in the liver (32, 33). GHR is structurally divided into three parts: the extracellular, transmembrane, and cytoplasmic domains. One molecule of GHR binds to one molecule of GH, the heterodimer, then interacts with another molecule of free GHR (34, 35). The homodimer of GHRs recruits and phosphorylates Janus kinase 2 (JAK2). JAK2 phosphorylation activates several signaling pathways, including the STAT5, ERK, and PI3K/Akt pathways (36). In addition, there is a class of soluble high-GH-affinity proteins called GHBPs, which share the sequence of the GHR extracellular domain. They can be generated by either alternative splicing of GHR pre-mRNA or cleavage of GHR protein (37–40). In addition to GHl, a unique GHR-like (GHRl) molecule has also been identified (30). GHRl has a structure similar to that of GHBP, which contains only an extracellular part. It shares approximately 30% sequence identity with the binding domain of vertebrate GHR. In addition, the locus of the *GHRL* gene (GenBank accession ID: LOC118431284) is closely linked to the *OXCT1* gene (LOC118431327) in the genome of the Florida amphioxus. This is similar to the vertebrate *GHR* gene. GHRl is mainly expressed in the hepatic caecum and gill, and has an expression pattern similar to that of vertebrate GHR. It can bind to both amphioxus GHl and zebrafish GH *in vitro*. Moreover, injection of GHRl affects the expression of IGF-I mRNA, which is stimulated by GH or amphioxus GHl in the zebrafish liver (30), indicating that GHRl interacts with GH and regulates its activity *in vivo*. These results suggest that GHRl is the binding partner of GHl.

It is worth mentioning that the amphioxus GHRl sequence cloned by Li et al. only encodes the extracellular domain. The corresponding predicted *GHRl* gene in the Florida amphioxus (LOC118431284) and *Branchiostoma belcheri* (LOC109467053) genomes also lacks the sequences encoding the transmembrane and cytoplasmic domains. It is noteworthy that a predicted gene encoding the transmembrane and cytoplasmic domains of type-I cytokine receptor (LOC118431288) is just adjacent to the *GHRl* gene in the Florida amphioxus. Perhaps a full-length *GHRl* gene was incorrectly noted as two separate genes owing to inaccurate genome assembly. The cloned truncated GHRl transcript might have resulted from alternative splicing resembling that of vertebrate GHBP. It is also possible that the GH signal is transduced *via* the receptor complex in the amphioxus. Some class-I helical cytokines (e.g., interleukin-6, ciliary neurotrophic factor and leukemia inhibitory factor) have multimeric receptors for signal transduction. Firstly, the ligand binds to a specific soluble receptor chain which might only contain the extracellular part. Then, the ligand-receptor heterodimer is transported to the target cell membrane, where it interacts with other transmembrane receptors. These receptors form a complex and trigger the signaling pathway (41). In ancient chordates such as the amphioxus, the original GHR might have just diverged from an ancestral form of type-I cytokine receptor which only contained an extracellular domain and needed to form multimeric receptors. The genes which encode the ligand-binding and signal transduction domains might further merge into a full-length *GHR* gene during evolution. The existence of full-length GHRl or other receptor partners of GHRl needs to be verified. The mechanism of GHl signal transduction also remains to be investigated.

## Amphioxus GHl and GHRl Illuminate the Origin of Vertebrate GH and GHR

GH belongs to the type-I helical cytokine superfamily (or four-helical cytokine superfamily). This superfamily includes GH, erythropoietin, interleukin, and ciliary neurotrophic factor. All the members comprise about 200 amino acids and have a characteristic four-helix bundle fold structure with an unusual topology (up-up-down-down). It is thought that all the type-I class cytokines evolved from a common ancestor through duplication and subsequent divergence (42–44). Among these members, prolactin (PRL) is evolutionarily and functionally related to GH (45–50). GH and PRL together constitute the GH family. The identification of the single *GH* gene in the agnathan, sea lamprey, indicated that *PRL* diverged from *GH* (51). It is thought that *GH* was generated by genome duplication and gene innovation during the evolution from invertebrates to vertebrates. However, it cannot be easily judged whether GH initially emerged in vertebrates. The study of Li et al. revealed the existence of a *GH-*like gene in basal chordates. The identification of a GHR-like molecule in the amphioxus further strengthens the hypothesis that GH may have originated from cephalochordates. It should be mentioned that invertebrate evolution into vertebrates occurred over a long period of time. Therefore, both GHl and GHRl in the amphioxus show primitive characteristics. For example, GHl shares low sequence identity with vertebrate GH, as well as other types of class-I helical cytokines. GHRl has an extended immunoglobulin-like domain in the N-terminal, which is characteristic of other types of class-I cytokine receptors such as some interleukin receptors. In conclusion, amphioxus GHl indicates that an ancestral form of a GH-like hormone emerged in cephalochordates. It might have transformed into GH during lengthy evolution through gene mutation and innovation rather than duplication of other genes.

GHR and PRL receptor (PRLR) are grouped into the GHR family and are thought to have a common ancestor. The single *GHRL* gene in the amphioxus strongly supports this hypothesis. It shares sequence identity with both GHRs and PRLRs. In addition, amphioxus GHl/GHRl makes the evolutionary order between GHl/GHR and PRL/PRLR clear. According to studies on vertebrates, it is difficult to identify which among GHl/GHR and PRL/PRLR arose in vertebrates first. Furthermore, both GHR and PRLR have been found in agnathan such as sea lamprey (52, 53). However, there is only one *GH* locus in the sea lamprey genome. Gong et al. suggested that sea lamprey GH might have a bidirectional effect and is capable of binding GHR or PRLR to activate different intracellular signals (53). Although GHRl shares slightly higher sequence identity with vertebrate PRLRs than GHRs, GHRl does not seem to be capable of transducing PRL signaling. Neither exogenous zebrafish PRL nor amphioxus GHl can increase the survival of the amphioxus in low salinity conditions (31). It can hence be inferred that PRL/PRLR and their downstream signal might not have diverged in cephalochordates. Therefore, GH/GHR emerged earlier in the evolution process.

## ILP and ILP Receptor in Amphioxus

IGF-I is the messenger that mediates the somatotrophic activity of GH. It is mainly expressed in the liver and induced by GH *via* activation of the JAK2-STAT5 signaling pathway (54–56). IGF-I synthesized by the liver plays mitogenic and metabolic roles in an endocrine manner. Circulating IGF-I interacts with IGF-IR located on the membranes of muscle and bone cells to activate the MAPK and PIK3/Akt pathways (57). IGF-I also acts *via* an autocrine/paracrine mechanism in some tissues (58–62). IGF-I belongs to the insulin superfamily, which includes insulin (INS) and IGF-II (63). They trigger overlapping signaling pathways and bind to receptors with a similar structure.

In the amphioxus genome, there is a single gene encoding an insulin-like peptide (ILP) which shows characteristics of both INS and IGFs. This ILP consists of 305 amino acids. The first 101 residues exhibit the features of pro-INS. The last 204 residues can be divided into D and E domains of IGF (64). The identification of ILP in basal chordates soundly demonstrated the existence of an ancestor of INS and IGF. Moreover, the single insulin-like peptide receptor (*ILPR*) gene was identified in the European amphioxus (*Branchiostoma lanceolatum*) by Pashmforoush et al. (65). The encoded receptor protein shares 48.6% sequence identity with the human INS receptor and 47.3% sequence identity with human IGF-IR. Autophosphorylation of this receptor could be stimulated with an amphioxus ILP analogue concentration of 10^-7^ M, indicating its high affinity for amphioxus ILP. Therefore, amphioxus ILP and ILPR reveal the origin of the INS/INSR system. Patton et al. proposed a model representing the evolution of the *INS/IGF* genes: an ancestral *INS/IGF-like* gene generated two genes by tandem duplication. They respectively evolved into *INS* and *IGF* by gene innovation. *IGF-I* and *IGF-II* diverged in the second round of genome duplication, whereas one of the duplicated *INS* genes was lost in the early Gnathostomata evolution (66).

Functionally, amphioxus ILP shows IGF-I-like mitogenic activity *in vitro*. Guo et al. cloned the orthologue of the *ILP* gene from Becher’s lancelet (*B. belcheri*). *In situ* hybridization and immunohistochemical staining showed that ILP is mostly enriched in the hepatic caecum and hind gut. The recombinant full-length ILP excluding the signal peptide showed mitogenic activity, which stimulated the proliferation of flounder gill cells (67). Liu and Zhang further proved that the recombinant artificially mature peptide of amphioxus ILP (IGF-MP, containing the B, C, A, and D domains of ILP) could bind to mouse IGFR and promote proliferation of mouse muscle cells in a dose-dependent manner. Western blotting showed that IGF-MP could activate the MAPK and PIK3/Akt signaling pathways, similar to human IGF-I (68). Moreover, the expression of ILP mRNA in the hepatic caecum could be induced by exogenous recombinant rat GH and amphioxus GHl (31, 67). These results indicate that amphioxus ILP and vertebrate IGF-I share features.

**Figure 1:**
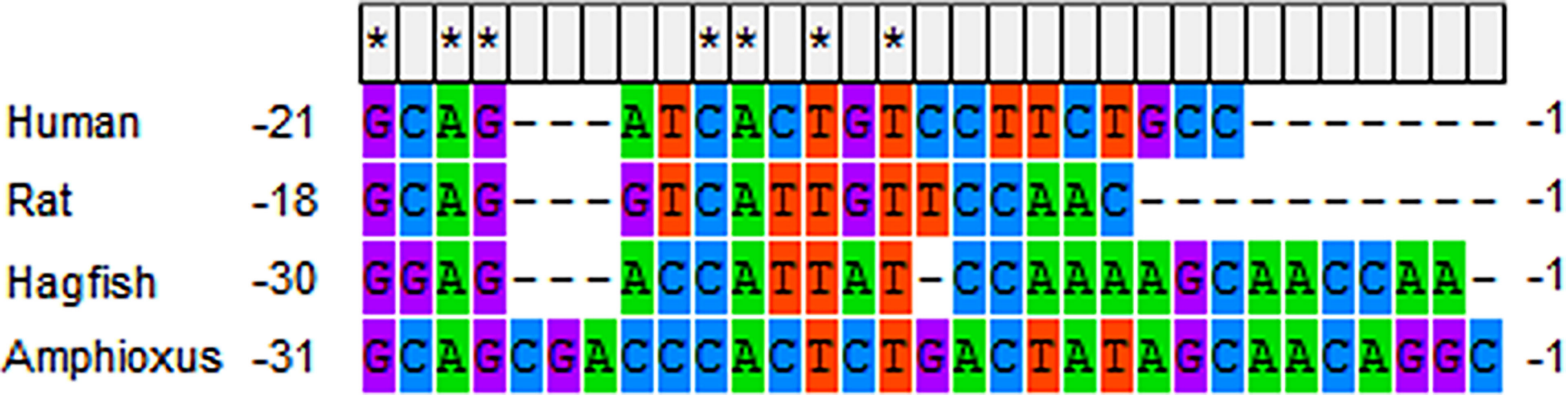
The glucose-responding *cis*-element in 5’ UTR of human *ins* mRNA (NM_000207.2), rat *ins* mRNA (NM_019130.2), hagfish *ins* mRNA (V00649.1) and amphioxus *ilp* mRNA (M55302.1). * indicates complete conservation.

It is uncertain whether ILP regulates metabolism. Although Guo et al. reported that injection of recombinant ILP could not reduce the blood glucose levels of diabetic mice induced by alloxan, the metabolic potency of ILP cannot be ruled out. Firstly, the B and A domains of IGF share about 47-57% and 44-51% sequence identity, respectively, with their homologous counterparts among the INS/IGF family members of the vertebrates (67), suggesting exogenous recombinant ILP might confer sensitivity onto mice. Secondly, the protokaryotic recombinant ILP was probably not forded or cleaved into the correct form to trigger metabolic signaling. Moreover, some characteristics of ILP and ILPR show underlying metabolic potency. For instance, amphioxus ILP is enriched in both the hepatic caecum and gut (67). In the amphioxus, the gut is not only the site of nutrient absorption, similar to the vertebrate intestine, but it is also partially homologous to the vertebrate pancreas. It had been observed that pancreatic hormone-producing cells are distributed in the amphioxus gut (69, 70). Elastase was also found to be specifically expressed in the amphioxus gut (71). The expression of ILP in the gut was similar to the expression of vertebrate INS in pancreatic islet β-cells. Moreover, the conserved glucose-response *cis*-element was located in the 5’ untranslated region (UTR) of amphioxus *ILP* (**Figure 1**), which is essential for responding to glucose stimulation (72). In addition, both human INS and IGF-I can induce autophosphorylation of amphioxus ILPR at a concentration of 10^-5^ M. The expression level of ILPR mRNA was approximately equal in the longitudinally divided four segments of a whole amphioxus (65). These are essential conditions of cell metabolism mediation by ILP. It is better to test the metabolic effect of ILP using the amphioxus as the experimental animal. It should especially be verified whether ILP can promote the accumulation of glycogen in the hepatic caecum (20).

## The Original IGFBP: IGF-Dependent or Independent?

IGFBP was initially identified as the carrier of IGFs in serum. It binds to and transports IGFs to the membranes of target cells. The half-life of IGFs significantly increases when they bind to IGFBPs (73–76). In addition to regulating the activity of IGF (77–81), IGFBP also exerts some IGF-independent functions such as transcription activation (82–88).

Six homologous IGFBPs encoded by different genes exist in mammals (89). These structurally related and multifunctional IGFBPs are thought to have duplicated and diverged from a common ancestor (90). This has been demonstrated by cloning the single *IGFBP* gene in *B.belcheri* (91). However, This IGFBP does not exhibit IGF affinity. In the IGF-binding domain of the vertebrate IGFBP sequence, there is a highly conserved R/KPLXXLL motif, which is critical for binding to IGF. In amphioxus IGFBP, the corresponding sequence of this motif is SPFRELL. After a single amino acid was mutated, this IGFBP was able to bind to IGF. Functionally, amphioxus IGFBP exerts transcription regulating activity. There are two nuclear localization motifs and an N-terminal in the transcription activation domain of amphioxus IGFBP sequence. This makes it localize in the nucleus and activate transcription.

To exclude the particularity of amphioxus IGFBP, the IGFBPs in urochordates and agnathans were considered. Urochordates and cephalochordates are the invertebrates which have the closest evolutionary relationship with vertebrates. Genomic scans showed that urochordates and cephalochordates are the only classes of invertebrates which have *IGFBP* genes in the genome. *Ciona intestinalis* is representative of urochordates. There are three putative genes (LOC100186815, LOC100182142, and LOC101242706) potentially encoding IGFBPs. One of these hypothetical IGFBPs does not have an IGF-binding domain due to incomplete genome assembly. Although the other two IGFBPs have IGF-binding domains, the critical motifs are ‘SNLIAIL’ and ‘YDAYGYL’, respectively, which are different from those of vertebrates. This implies that they might not bind to IGF either. In agnathans, IGFBP could interact with IGF. Sea lamprey IGFBP3 has a conserved ‘RPLQALL’ motif. It has been demonstrated to have IGF affinity and play an IGF-dependent role (92). It is probable that IGFBP was initially independent of the GH/IGF system in invertebrates. It gained IGF affinity during evolution. It is also possible that the original IGFBP could bind to ILP, unlike vertebrate IGF. The interaction between amphioxus IGFBP and ILP needs to be further examined.

## Conclusion and Perspective

**Figure 2:**
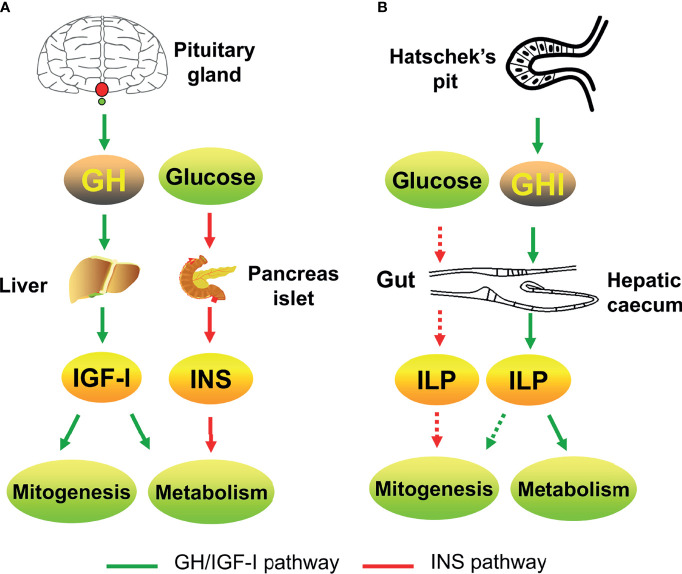
The GH/IGF systems of vertebrates [e.g., human; **(A)**] and amphioxus **(B)** involve in growth and metabolism. Green arrow indicates the GH/IGF pathway; red arrow indicates the insulin pathway. The solid line indicates confirmed parts; the dotted line indicates putative parts.

The GH/IGF system is the endocrine axis that connects the pituitary and liver. It regulates the postnatal growth of vertebrates. The origin of the GH/IGF system has remained unknown for a long time. The basal chordate, amphioxus, has structures homologous to the vertebrate pituitary and liver. The identification of *GH/IGF*-like genes in the amphioxus has not only revealed the origin of the GH/IGF system gene families but also indicated that the vertebrate-like endocrine axis emerged in invertebrates (**Figure 2**). This largely strengthens the evolutionary position of the amphioxus and deepens the understanding of the animal evolution process. In particular, the discovery of amphioxus GHl and ILP, as well as their receptors, reinforces the homologies between the amphioxus Hatschek’s pit and vertebrate pituitary as well as the hepatic caecum and liver (**Table 1**).

**Table 1:** The identified members of GH/IGF system in amphioxus.

Name	Expression	characters	GeneBank ID	Reference
Growth hormone-like	Widely expressed, enriched in Hatschek’s pit and gill	Somatotrophic activity, osmoregulatory activity, binds to GHR, induces expression of IGF-I and ILP	KJ735508	(30, 31)
Growth hormone receptor-like	Widely expressed, enriched in hepatic caecum and gill	Binds to GH	KJ735509	(30)
Insulin-like peptide	Enriched in hepatic caecum and gut	Induced by GH, mitogenic activity	M55302	(64, 67, 68)
			EU420069	
Insulin-receptor like	Widely expressed	Binds to ILP, INS, and IGF-I, activated by them	S83394	(65)
Insulin-like growth factor binding protein	Expressed throughout early development	Nucleus localization, transcription activation	FJ971406	(91)

Owing to the lack of an amphioxus cell line, the downstream signaling pathways of the amphioxus GH/IGF system are unclear. The rapid development of single-cell sequencing and proteomics techniques would be beneficial to the separation of target cells and identification of the intracellular messengers.

GH belongs to the type-I helical cytokine superfamily. In addition to that of the GH-like hormone, there are several genes related to type-I helical cytokines in the amphioxus genome. The putative proteins encoded by these genes are quite divergent from the known vertebrate type-I helical cytokines, implying that they might be ancestors, which would be further divided into other classes of type-I helical cytokines. Moreover, no type-I helical cytokine gene has been detected in the genomes of urochordates, including acorn worms and sea squirts. In this regard, although some analysis had suggested that urochordates and vertebrates were more likely sister clades, cephalochordates are the only class of invertebrates that have been demonstrated to have a vertebrate-like endocrine system. It seems that the evolutionary relationship between cephalochordates, urochordates, and vertebrates is more complex than once thought. On the other hand, the functions of other ancient type-I helical cytokines and the origin of this superfamily remain undetermined.

In addition, some questions remain unsolved. For example, what is the complete signaling pathway of GHl in the amphioxus? Is there a full-length GHRl in the amphioxus? Does ILP have a metabolic effect? Clarifying these points will help establish a more integrated GH/IGF system of the amphioxus.

## Author Contributions

ML confirms being the sole contributor of this work and has approved it for publication.

## Funding

This work was supported by the National Natural Science Foundation of China (grant number 81900259).

## Conflict of Interest

The author declares that the research was conducted in the absence of any commercial or financial relationships that could be construed as a potential conflict of interest.

## Publisher’s Note

All claims expressed in this article are solely those of the authors and do not necessarily represent those of their affiliated organizations, or those of the publisher, the editors and the reviewers. Any product that may be evaluated in this article, or claim that may be made by its manufacturer, is not guaranteed or endorsed by the publisher.
